# Phenotypic and genotypic variations within a single bacteriophage species

**DOI:** 10.1186/1743-422X-8-134

**Published:** 2011-03-23

**Authors:** Pieter-Jan Ceyssens, Thea Glonti, ndrew M Kropinski, Rob Lavigne, Nina Chanishvili, Leonid Kulakov, Nino Lashkhi, Marina Tediashvili, Maya Merabishvili

**Affiliations:** 1 Laboratory of Gene Technology (LoGT), Katholieke Universiteit Leuven, Kasteelpark Arenberg 21 bus 2462, B-3001 Leuven, Belgium; 2 Eliava Institute of Bacteriophage, Microbiology and Virology (EIBMV), 3 Gotua Street, 0160 Tbilisi, Georgia; 3 Laboratory for Foodborne Zoonoses, Public Health Agency of Canada, 110 Stone Road West, Guelph, ON, N1G 3W4, Canada; 4 Department of Molecular & Cellular Biology, University of Guelph, Guelph, ON, N1G 2W1, Canada; 5 School of Biological Sciences, The Queen's University of Belfast, Medical Biology Centre, 97 Lisburn Road, Belfast BT9 7BL, Northern Ireland; 6 Laboratory for Molecular and Cellular Technology (LabMCT), Burn Wound Center, Queen Astrid Military Hospital, Bruynstraat 1, 1120 Brussels, Belgium

## Abstract

**Background:**

Although horizontal gene transfer plays a pivotal role in bacteriophage evolution, many lytic phage genomes are clearly shaped by vertical evolution. We investigated the influence of minor genomic deletions and insertions on various phage-related phenotypic and serological properties.

**Findings:**

We collected ten different isolates of *Pseudomonas aeruginosa *bacteriophage ϕKMV. All sequenced genomes (42-43 kb, long direct terminal repeats) are nearly identical, which intuitively implied strongly similar infections cycles. However, their latent periods vary between 21 and 28 minutes and they are able to lyse between 5 and 58% of a collection of 107 clinical *P. aeruginosa *strains. We also noted that phages with identical tail structures displayed profound differences in host spectra. Moreover, point mutations in tail and spike proteins were sufficient to evade neutralization by two phage-specific antisera, isolated from rabbits.

**Conclusion:**

Although all analyzed phages are 83-97% identical at the genome level, they display a surprisingly large variation in various phenotypic properties. The small overlap in host spectrum and their ability to readily escape immune defences against a nearly identical phage are promising elements for the application of these phages in phage therapy.

## 

The potential use of lytic bacteriophages as alternative therapeutic agents against antibiotic-resistant bacteria has been widely documented [1-3]. This renewed interest in phage therapy resulted in human clinical trials [4,5] and a considerable rise of commercial interest [6]. Potential downsides of phage applications, i.e. their relatively narrow host range and emerging bacterial resistance, are often countered by the proposed use of mixtures (cocktails) of phages targeting different hosts and/or host receptors. A popular argument in favour of these cocktails is the sheer abundance of phages in nature (10^31 ^particles worldwide) and the ease of isolating new phages infecting phage-resistant bacteria [7].

In spite of their ubiquitous character, numerous studies suggest the existence of only a limited number of virulent phage 'types' targeting a specific bacterial species. For example, despite decades of intensive research, only 17 and 13 distinct phage species infecting the model organisms *Escherichia coli *and *Pseudomonas aeruginosa *are known [8,9]. Nearly all newly isolated lytic phages infecting these organisms cluster within existing species and carry genomes which are often over 90% identical at the nucleotide level [10-12]. As this limited global diversity might hamper the composition of truly diverse phage cocktails, we were interested in the impact of these subtle "intraspecies" genomic insertions and deletions on infection-related and serological properties of lytic bacteriophages.

As a model species, we choose the virulent *P. aeruginosa *phage ϕKMV which resembles the classic coliphage T7 in morphology and overall genome architecture [13]. It was also the first known T7-related phage encoding a single-subunit RNA polymerase gene downstream its DNA metabolism genes instead of in the early genomic region [13]. Up to now, three phages infecting *P. aeruginosa *(LKD16, ϕKF77 and LUZ19) have been reported to be 83-90% identical to ϕKMV at the nucleotide level [14-16]. In addition, phage LUZ2 was reported to be closely related to ϕKMV based on *de novo *analysis of structural phage proteins [12].

Phages PT2, PT5, PT6, PNC101 and PNM were isolated from Mtkvari River in Georgia [17] and added to our existing collection of ϕKMV-related isolates (Table 1). We also included the more distantly related *Pseudomonas *phage LKA1 in this analysis; LKA1 shares significant sequence similarity for only 48% of its gene products but has a conserved functional genome organization and gene order [14]. With the exception of LKA1 which forms small plaques (1 mm), all of these phages produce large (4-10 mm diameter) clear plaques. As members of the family *Podoviridae*, these viruses possess icosahedral heads (diameter of 50-60 nm) and 10-12 nm noncontractile tails. With the exception of LKA1, all the analyzed phages lyse *P. aeruginosa *cells 21 to 28 minutes after infection at 37°C upon the release of between 116 and 166 newly produced virions (Table 1).

**Table 1:** Overview of ϕKMV-related bacteriophages included in this study, all infecting *P. aeruginosa*.

Phage	Isolated in	Genome (bp)	# ORFs	DTR length	GC (%)	ϕ**KMV identity**^**a**^	Accession number	**K**_**adsorption **_**(ml/min)**^**b**^	Latent period (min)	Burst size (PFU/cell)	**K**_**APS/PT5**_^**c**^	**K**_**APS/PNC101**_^**c**^
**ϕKMV**	Moscow, Russia	42,519	52	414	62.3	100	NC_005045	1.6 × 10^-8^	28	116	259	849
**LKD16**	Leuven, Belgium	43,200	54	428	62.6	83	NC_009935	-	27	120	87.5	-
**LUZ19**	Leuven, Belgium	43,548	54	472	62.2	89	NC_010326	1.2 × 10^-7^	22	122	-	-
**ϕKF77**	Moscow, Russia	43,152	53	454	62.8	90	NC_012418	1.5 × 10^-8^	23	129	56.2	-
**PNM**	Tbilisi, Georgia	42,721	51	422	62.3	94	-	ND	ND	ND	-	23
**PT2**	Tbilisi, Georgia	42,961	54	488	62.0	97	NC_011107	9.2 × 10^-9^	21	116	737	51
**PT5**	Tbilisi, Georgia	42,954	52	413	62.0	95	NC_011105	7.3 × 10^-9^	21	134	598	679
**PT6**	Tbilisi, Georgia	ND	ND	ND	ND	ND	-	2.1 × 10^-8^	25	166	257	857
**PNC101**	Tbilisi, Georgia	ND	ND	ND	ND	ND	-	ND	ND	ND	60	753
**LUZ2**	Leuven, Belgium	ND	ND	ND	ND	ND	-	ND	ND	ND	270	-
**LKA1**	Leuven, Belgium	41,593	56	298	60.9	ND	NC_009936	3.9 × 10^-9^	41	225	-	-

^a ^Identity with ϕKMV, expressed as % identical nucleotides according to the Stretcher algorithm (EMBOSS suite).

^b ^The adsorption constant k was calculated from the unabsorbed phage at a multiplicity of infection of 1:1 by applying the formula k_a _= 2.3/Bt log P_0_/P, where P_0 _is initial number of phage, P is number of unabsorbed phage particles after time t and B is number of bacteria (4). ND, not determined.

^c ^Serological neutralization assay using PT5 and PNC101 APS. Dilutions (1/50- 1/1000) of APS were mixed with 10^6 ^bacteriophage, samples were titered after different incubation times (5, 15 and 30 min) at 37°C. K, rate constant of neutralization, defined as k = 2.3 D/t logP_0_/P, where P_0 _- count of bacteriophage plaques at zero time, P - count of bacteriophage plaques after t time, D - dilution of APS.

Infectivity screens on various outer membrane mutants of *P. aeruginosa *unambiguously identified the necessity for Type IV pili for successful infection of all ϕKMV-like phages except for LKA1, which depends on *alg*C expression. The presence/absence of bacterial flagella (encoded by *fli*C) has no influence on phage infection (data not shown). Despite this common type IV pili dependence, subsequent screens on a large collection of 114 clinical *P. aeruginosa *strains showed profound differences in host range. Between 18 (ϕKMV) and 62 (PT6) *P. aeruginosa *strains were sensitive to phage infection, while the more distantly related phage LKA1 can only lyse six strains from our collection (Figure 1A, Additional file 1). This observation pointed out variations in secondary adsorption mechanisms and intracellular development, and provoked interest in the genome sequences of these new ϕKMV-related isolates.

**Figure 1:**
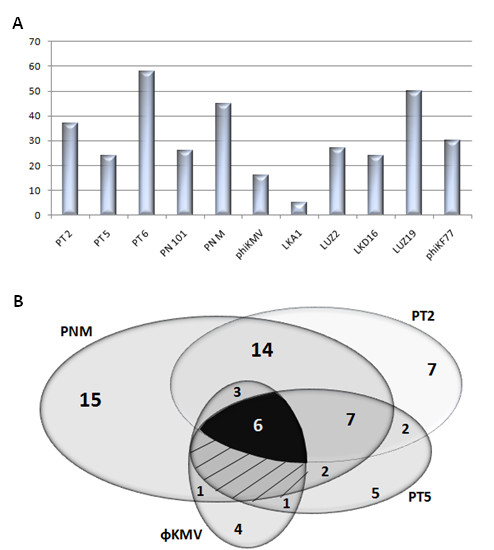
**Host range analysis of the ϕKMV-like viruses**. **A**. Host range analysis of 'phiKMV-like viruses' using a collection of 114 clinical *P. aeruginosa *strains. In this assay, 10^6 ^pfu were spotted on a bacterial lawn and checked for lysis. See additional files for more information on the *P. aeruginosa *strains and for detailed spectra. **B**. Host range comparison of four phages displaying >94% nucleotide identity throughout their genomes, stating the number of strains which are uniquely or commonly lysed by PNM, PT2, PT5 and phiKMV.

Using traditional Sanger dideoxy methods for PNM and 454 pyrosequencing for PT2 and PT5, the complete genome sequences of these phages were determined (Table 1, Figure 1). All three phages display high nucleotide similarity (94-97% identity) to ϕKMV, except for a 382 bp region located between ORF3 and 4 (Figure 1), which was earlier associated with a localized single-stranded nick on the non-coding strand in the related phages LUZ19 and ϕKF77 [16]. As ϕKMV is the only phage of this species which has lost this nick, it is most probably a more recent representative of this phage species.

When comparing these newly sequenced genomes to all known ϕKMV-related isolates at the protein level, the extraordinary conservation throughout their genomes is evident. With the exception of occasional insertions (*e.g*., the unrelated ORFs 17.1 in LKD16 and ϕKF77) and deletions (e.g., ORF2 in ϕKF77, ORF20 in PNM), the genomes have been stably maintained over time and location. The only phage encoding an additional structural protein is LUZ19 (gp49), which might cause subtle differences in virion. The tail fiber regions of ϕKMV, PT2 and PNM (and of the partially sequenced phage PT6) are 99.5-100% identical, strongly suggesting identical reception/adhesion mechanisms. A similar conservation of tail genes is also present between LUZ19 and ϕKF77 (Figure 2). In contrast, these genes are far less conserved in PT5 (25-52% amino acid identity to ϕKMV), whose tail fibers are more related to phage LKD16 (61-94% amino acid identity). In contrast to clear differences in the tail fibers genes of PT5 and PNM, their early coding region is completely identical (Figure 2). Intriguingly, the fact that ϕKMV, PT2, PNM and PT5 have nearly identical virions and/or early regions does not correlate to their respective host spectra. As shown in Figure 1B, only six *P. aeruginosa *strains are lysed by all four phages and 30 strains can only be targeted by a single phage.

**Figure 2:**
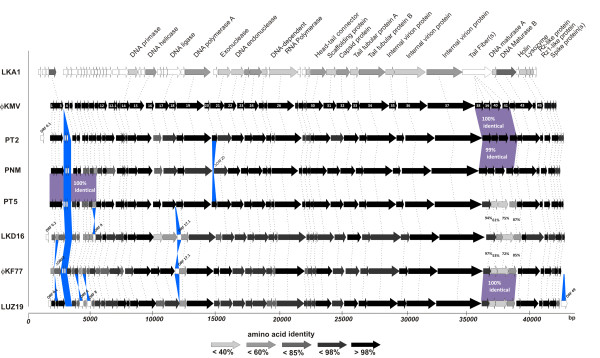
**Comparative genomic analysis of the ϕKMV-like viruses**. The predicted open reading frames of all sequenced ϕKMV-like phages are shown. Their amino acid identity to the corresponding ORF of ϕKMV is indicated in different shades of grey. ORFs unique to each phage are not colored, while predicted functions of the annotated genes are indicated on top. Functional equivalent genes are connected with broken lines. Gene annotation was performed as described elsewhere (Ceyssens *et al*., 2006).

In a final step, the serological relatedness of these phages was studied by using a classical cross neutralization method [18]. Two anti-phage sera (APS) were obtained by a two-step immunization of three Swiss white rabbits with phages PT5 and PNC101. Each rabbit was injected intramuscularly with a 1:1 (vol/vol) mixture of phage (10^10 ^pfu/ml) and adjuvant (Freund's complete adjuvant, Difco). One month later, a secondary immunization was performed by injecting phage (1 ml, 10^11 ^pfu/ml) without adjuvant. After two weeks, blood was taken from the left ventricle of the heart. This blood was allowed to clot, the APS was filtered (0.45 μm pore size) and stored at 4°C. Subsequent neutralization assays with these APS showed significant variation in serological response among the tested phages, as only ϕKMV and PT5 were efficiently neutralized by both antisera (Table 1). Phages LKA1 and LUZ19 were not neutralized to any significant degree by either APS, while the inactivation of LKD16, ϕKF77 and PNM was at least tenfold less efficient than of the phages used for the APS production.

When looking into these results in more detail, some surprising observations were made. One would expect that neutralizing antibodies primarily function through interaction with capsid and/or tail (fibers) proteins of these phages. It is therefore curious that PT2 but not PNM is neutralized by APS_PT5_, since the structural proteins of PT2 and PNM are all >99.5% identical (Figure 2). Comparing the genomes of these three phages, the only non-silent structural mutations present in PT2 and PT5 in comparison to PNM are found in the tail tubular proteins A (P_20_-L_20_, K_55_-R_55_, T_102_-P_102, _R_115_-H_115_) and B (D_26_-G_26, _T_111_-N_111, _V_142_-I_142_, A_298_-G_298_, G_329_-D_329_, R_363_-S_363_, A_644-_V_644_, P_660_-S_660_, A_784_-V_784_). These amino acids substitutions are conserved in ϕKMV, explaining the sensitivity of this phage for APS_PT5_. A similar finding was observed using the anti-phage serum produced against the non-sequenced phage PNC101. This serum was over tenfold more effective in neutralizing ϕKMV and PT5 than PT2 and PNM, despite 100% identity of their capsid and connector proteins (Table 1, Figure 2). This time, the difference might be correlated to four amino acid substitutions in the spike protein (gp48) of PT2 and PNM.

## Discussion

Although horizontal gene transfer plays a pivotal role in bacteriophage evolution, many lytic phage genomes are clearly shaped by vertical evolution. The high level of genomic conservation (83-97% identity) observed within the 'phiKMV-like viruses' is a common theme among virulent phages infecting the same bacterial host; for example, the genome of Roseophage SIO1 was found to be completely conserved in isolates sampled over many years in various places [11]. Typically, strongly related phages attach to the same receptor and carry out a highly comparable infection cycle. At first glace, this is indeed the case for all analyzed ϕKMV-like viruses. They are all pili-dependent and quickly lyse their host upon the release of up to 166 newly produced particles.

During this study we noticed that small "intraspecies" genomic variations can have essential phenotypic consequences towards the applications of these phages in therapeutic settings. First of all, only limited overlaps in host spectrum exist between isolates with identical tail fibers and/or early genome regions, implying that minor genomic changes can cause a significant shift in infectivity range. Although these differences could be attributed to the evasion of CRISPR repeats [19], a recent survey did not find a single spacer matching a lytic bacteriophage sequence in 122 clinical *P. aeruginosa *strains [20]. Alternatively, small point mutations could help in the evasion of host restriction-modification systems [21]. In any case, this illustrates the versatility of a phage genome to evade host defenses.

Moreover, phage therapy seems not be hindered by the adaptive immune system, since minor variations in tail/capsid structures seem sufficient to evade antibody binding. This confirms observations made by Vitiello and colleagues [22], who reported a long-circulating mutant of phage λ which carried only a single mutation in the major capsid protein. As phages have a virtually unlimited potential of subtly modifying their virion, the potential is present to quickly overcome host immune responses and bacterial resistance during phage therapy.

## Competing interests

The authors declare that they have no competing interests.

## Authors' contributions

PJC, RL and AMK performed comparative genomics experiments. TG, NC, LK, NL and MT isolated the phages and performed serological and infection-related experiments. MM coordinated these and performed host range studies. PJC wrote the manuscript. All authors read and approved the final manuscript.

## Supplementary Material

Additional file 1 **Detailed host spectra of the analyzed ϕKMV-like viruses**. Host range analysis of 'phiKMV-like viruses' using a collection of 114 clinical *P. aeruginosa *strains. In this assay, 10^6 ^pfu were spotted on a bacterial lawn and checked for lysis. The file contains information on the origin of the bacterial strains (country/sampling site) and the level of lysis caused by addition of the phage. Click here for file
