# Supplementary material for: Phenotypic and genotypic variations within a single bacteriophage species
Source: Virol J. 2011 Mar 23;8:134. doi: 10.1186/1743-422X-8-134 (PMC3072928; doi:10.1186/1743-422X-8-134)
Supplement: Additional file 1 — Detailed host spectra of the analyzed ϕKMV-like viruses. Host range analysis of 'phiKMV-like viruses' using a collection of 114 clinical P. aeruginosa strains. In this assay, 106 pfu were spotted on a bacterial lawn and checked for lysis. The file contains information on the origin of the bacterial strains (country/sampling site) and the level of lysis caused by addition of the phage. [file 1743-422X-8-134-S1.DOCX]

**SUPPLEMENTARY DATA**

Supplemental data on 114 isolated of *P. aeruginosa* used in this study.

| ***Pseudomonas aeruginosa*** | | | | **Phage^a^** | | | | | | | | | | |
| --- | --- | --- | --- | --- | --- | --- | --- | --- | --- | --- | --- | --- | --- | --- |
| ***strain*** | ***Year*** | ***Origin^a^*** | ***Sample^b^*** | **PT 2** | **PNM** | **phiKMV** | **PT 5** | **PT 6** | **PNC101** | **LKA1** | **LUZ2** | **LKD16** | **LUZ19** | **ɸKF77** |
| BU007 | 1997 | Budapest (Hun) | Burn Wound | - | - | - | - | - | - | - | - | - | - | - |
| W15DEC11 | 2001 | Brussels (Bel) | River Water | cl | ol | cl | cl | cl | cl | - | cl | cl | cl | cl |
| PA01 | 1955 | Melbourne (Aus) | Wound | - | - | - | - | - | - | cl | - | - | cl | - |
| W15DEC14 | 2001 | Brussels (Bel) | River Water | - | - | ol | ol | sp | ol | - | - | - | scl | scl |
| LMG14084 | 1964 | Bucarest (Rom) | Water | - | - | ol | - | - | - | - | scl | ol | - | scl |
| TUD47 | 1998 | Tunis (Tun) | Ascites | cl | cl | cl | cl | cl | cl | - | cl | cl | cl | cl |
| BR667 | 1998 | Brussels (Bel) | Burn Wound | - | - | - | - | - | - | - | - | - | - | - |
| C | 1989 | Hannover (Ger) | Sputum | - | - | ol | - | ol | - | - | - | - | - | scl |
| BR257 | 1997 | Brussels (Bel) | Plant Rhizosphere | cl | cl | - | - | cl | sp | - | scl | scl | - | scl |
| LMG5031 | 1961 | Puerto Rico | Chinese Evergreen | - | - | - | - | scl | - | - | scl | scl | scl | - |
| IS573 | 1997 | Istanbul (Tur) | Burn Wound | - | ol | - | - | - | - | - | - | - | - | - |
| BR670 | 1998 | Brussels (Bel) | Sputum | - | ol | - | - | - | - | - | - | - | - | - |
| LO049 | 1996 | London (UK) | Burn Wound | - | - | - | - | - | - | - | - | - | - | - |
| BR231 | 1997 | Brussels (Bel) | Bathroom Burn Unit | - | - | scl | - | - | - | - | cl | cl | ol | scl |
| ATCC27853 | 1971 | Boston (USA) | Blood | ol | ol | - | - | ol | - | - | cl | ol | scl | scl |
| PA6 | 1985 | Brussels (Bel) | Urine | ol | - | - | ol | - | - | - | - | - | - | - |
| C13 | 1985 | Hannover (Ger) | Sputum | ol | ol | ol | - | cl | ol | - | cl | cl | scl | cl |
| PHDW6 | 1993 | Tacloban City (Ph) | Wound | sp | ol | - | - | scl | ol | - | scl | scl | scl | scl |
| Tu863 | 1998 | Tunis (Tun) | Ear | ol | ol | ol | sp | ol | ol | - | cl | cl | cl | cl |
| SG17M | 1992 | Ruhr river (Ger) | Sputum | ol | ol | cl | - | cl | ol | - | cl | cl | cl | cl |
| BR735 | 1998 | Brussels (Bel) | Ear | - | - | - | - | - | - | - | ol | - | - | scl |
| Aa245 | 1997 | Aachen (Ger) | Burn Wound | - | - | - | - | - | - | - | - | - | - | - |
| LW1048 | 2001 | Lwiro (Con) | Blood | cl | ol | - | ol | cl | - | - | - | - | - | - |
| PA023 | 1998 | Karachi (Pak) | River Water | sp | ol | ol | sp | sp | - | - | - | - | scl | scl |
| BR217 | 1997 | Brussels (Bel) | Sink Burn Unit | ol | ol |  | - | ol | - |  | scl | ol | cl | scl |
| SO92 | 1997 | Sofia (Bul) | Burn Wound | - | ol | - | - | - | - | - | - | - | - | - |
| W15DEC8 | 2001 | Brussels (Bel) | River Water | - | - | - | - | - | - | - | - | - | - | cl |
| 0107 2844 | 2001 | Ghent (Bel) | Sputum | - | - | - | - | - | - | - | - | - | cl | - |
| 0107 2896B | 2001 | Ghent (Bel) | Sputum | ol | scl | ol | ol | cl | sp | - | ol | - | ol | - |
| 0108 5622 | 2001 | Ghent (Bel) | Sputum | - | cl | - | - | - | cl | - | - | - | - | - |
| 0109 2348 | 2001 | Ghent (Bel) | Sputum | ol | sp | - | ol | ol | sp | - | - | - | - | - |
| 010917-15 | 2001 | Ghent (Bel) | Sputum | sp | - | cl | sp | cl | - | - | cl | ol | ol | cl |
| 0102 1495A | 2001 | Ghent (Bel) | Sputum | ol | ol | - | ol | cl | ol | - | - | - | - | - |
| 0106 2918 | 2001 | Ghent (Bel) | Sputum | - | ol | - | - | cl | ol | - | - | - | ol | - |
| 0110 0840 | 2001 | Ghent (Bel) | Sputum | - | - | - | - | cl | - | - | - | - | ol | - |
| 0110 0716 | 2001 | Ghent (Bel) | Sputum | - | cl | - | - | - | - | - | - | - | - | - |
| 0110 0826 | 2001 | Ghent (Bel) | Sputum | cl | - | - | - | cl | - | sp | - | - | - | - |
| 0108 1953 | 2001 | Ghent (Bel) | Sputum | - | - | - | - | cl | - | sp | - | - | ol | - |
| 0110 3252 | 2001 | Ghent (Bel) | Sputum | - | - | - | - | ol | - | - | scl | - | - | - |
| 0102 1406 | 2001 | Ghent (Bel) | Sputum | ol | ol | - | - | cl | ol | - | - | - | - | - |
| 0102 1426 | 2001 | Ghent (Bel) | Sputum | - | - | - | - | - | - | - | - | - | - | - |
| 0102 4218 | 2001 | Ghent (Bel) | Sputum | - | - | - | - | - | - | - | - | - | - | - |
| 0102 5041 | 2001 | Ghent (Bel) | Sputum | - | - | - | - | - | - | - | - | - | scl | - |
| 0102 6048 | 2001 | Ghent (Bel) | Sputum | - | - | - | - | ol | - | - | - | - | - | - |
| 010108-06 | 2001 | Ghent (Bel) | Sputum | - | - | - | - | - | - | - | - | - | - | - |
| 0102 6027 | 2001 | Ghent (Bel) | Sputum | ol | ol | - | sp | ol | ol | - | - | - | scl | - |
| 0103 1519 | 2001 | Ghent (Bel) | Sputum | - | - | - | cl | cl | - | - | - | - | ol | - |
| 0103 1521 | 2001 | Ghent (Bel) | Sputum | - | - | - | - | cl | - | - | - | - | ol | - |
| ESP01 | 1993 | CESPA (Bel) | Unknown | cl | ol | - | - | cl | - | - | - | - | - | scl |
| ESP02 | 1993 | CESPA (Bel) | Unknown | - | ol | - | ol | sp | - | scl | cl | cl | ol | cl |
| ESP06A | 1993 | CESPA (Bel) | Unknown | ol | ol | - | - | scl | - | - | scl | scl | cl | cl |
| ESP07 | 1993 | CESPA (Bel) | Unknown | - | scl | - | - | ol | - | - | - | - | - | - |
| ESP11 | 1993 | CESPA (Bel) | Unknown | - | sp | - | - | - | - | - | - | - | cl | - |
| ESP14 | 1993 | CESPA (Bel) | Unknown | - | - | - | - | - | - | - | - | - | cl | - |
| ESP15 | 1993 | CESPA (Bel) | Unknown | ol | ol | scl | - | ol | - | - | scl | scl | ol | scl |
| ESP16 | 1993 | CESPA (Bel) | Unknown | - | - | - | - | - | - | - | - | - | - | - |
| ESP21 | 1993 | CESPA (Bel) | Unknown | - | - | - | ol | - | - | - | scl | scl | scl | scl |
| ESP23 | 1993 | CESPA (Bel) | Unknown | - | - | - | - | ol | - | - | - | - | - | - |
| ESP25 | 1993 | CESPA (Bel) | Unknown | - | ol | ol | - | ol | - | - | scl | ol | cl | scl |
| ESP31 | 1993 | CESPA (Bel) | Unknown | ol | cl | - | - | cl | - | - | - | - | - | ol |
| 011214 1668 | 2001 | Ghent (Bel) | Sputum | - | ol | - | - | - | - | - | - | - | cl | - |
| 011213 1371 | 2001 | Ghent (Bel) | Sputum | ol | ol | - | - | cl | - | - | - | - | scl | - |
| 99 06 2105 | 1999 | Ghent (Bel) | Sputum | - | cl | - | - | - | cl | - | - | - | - | - |
| 99 07 0393 | 1999 | Ghent (Bel) | Sputum | - | scl | - | - | sp | - | - | - | scl | - | - |
| 020208 1814 | 2002 | Ghent (Bel) | Sputum | - | - | - | cl | sp | - | ol | - | - | ol | - |
| 020124 1990 | 2002 | Ghent (Bel) | Sputum | ol | ol | - | ol | cl | ol | ol | - | - | ol | - |
| 020213 1916 | 2002 | Ghent (Bel) | Sputum | sp | - | - | sp | cl | ol |  | - | - | ol | - |
| 0102 1370 | 2001 | Ghent (Bel) | Sputum | - | - | - | - | - | - | - | - | - | - | - |
| 0102 1372 | 2001 | Ghent (Bel) | Sputum | - | - | - | - | - | - | - | - | - | - | - |
| 010402-04 | 2001 | Ghent (Bel) | Sputum | - | - | - | - | - | - | - | - | - | cl | - |
| 010402-04 | 2001 | Ghent (Bel) | Sputum | - | - | - | - | - | - | - | - | - | cl | - |
| 020306 1731 | 2002 | Ghent (Bel) | Sputum | - | - |  | - | - | - |  |  |  | cl | - |
| 020306 1845 | 2002 | Ghent (Bel) | Sputum | - | - | - | - | - | - | - | - | - | - | - |
| 0102 1373 | 2001 | Ghent (Bel) | Sputum | - | - | - | - | - | - | - | - | - | scl | - |
| 99 04 5901 | 1999 | Ghent (Bel) | Sputum | - | sp | - | sp | cl | - | - | - | - | - | - |
| 99 04 6389 | 1999 | Ghent (Bel) | Sputum | - | - | - | - | - | - | - | - | - | scl | - |
| 99 04 6061 | 1999 | Ghent Bel) | Sputum | sp | - | - | - | ol | sp | - | - | - | - | - |
| 99 04 6420 | 1999 | Ghent (Bel) | Sputum | sp | - | - | - | sp | - | - | - | - | - | - |
| 99 05 1954 | 1999 | Ghent (Bel) | Sputum | cl | - | - | - | - | - | - | - | - | scl | - |
| 011022-12 | 2001 | Ghent (Bel) | Sputum | - | sp | - | - | cl | sp | - | - | - | - | - |
| 020402-18 | 2002 | Ghent (Bel) | Sputum | ol | sp | - | cl | cl | sp | - | - | - | cl | - |
| 010430-14 | 2001 | Ghent (Bel) | Sputum | - | - | - | sp | - | - | - | - | - | cl | - |
| 011126-16 | 2001 | Ghent (Bel) | Sputum | ol | sp | - | - | cl | ol | - | ol | - | ol | ol |
| 011203-13 | 2001 | Ghent (Bel) | Sputum | cl | cl | - | cl | cl | cl | - | cl | scl | ol | cl |
| 020325-14 | 2002 | Ghent (Bel) | Sputum | - | ol | - | - | cl | ol | - | - | - | - | - |
| 010326-20 | 2001 | Ghent (Bel) | Sputum | - | - | - | - | - | - | - | - | - | ol | - |
| 0102 1427 | 2001 | Ghent (Bel) | Sputum | - | - | - | - | cl | - | - | - | - | - | - |
| 020606 2051 | 2002 | Ghent (Bel) | Sputum | - | - | - | - | - | - | - | - | - | - | - |
| H92 10531 | 1992 | Ghent (Bel) | Unknown | ol | ol | - | - | cl | - | - | cl | scl | scl | scl |
| 020702-17 | 2002 | Ghent (Bel) | Sputum | ol | - | - | - | ol | - | - | - | - | - | - |
| 020716 1981 | 2002 | Ghent (Bel) | Sputum | - | - | - | - | - | - | - | - | - | - | - |
| S90 02441 | 1990 | Ghent (Bel) | Sputum | ol | - | - | - | ol | - | - | - | - | cl | - |
| 020722-03 | 2002 | Ghent (Bel) | Sputum | cl | cl | cl | cl | cl | cl | - | cl | cl | ol | cl |
| 020729-05 | 2002 | Ghent (Bel) | Sputum | cl | sp | - | - | cl | - | - | - | - | - | - |
| 020916-13 | 2002 | Ghent (Bel) | Sputum | - | - | - | - | - | - | - | - | - | - | - |
| 0102 1663 | 2001 | Ghent (Bel) | Sputum | cl | - | scl | cl | cl | scl | - | scl | scl | cl | scl |
| 0102 1560B | 2001 | Ghent (Bel) | Sputum | sp | ol | ol | cl | scl | ol | - | scl | ol | ol | cl |
| 0102 1901A | 2001 | Ghent (Bel) | Sputum | - | - | ol | - | - | - | - | cl | ol | - | ol |
| 021025-26 | 2002 | Ghent (Bel) | Sputum | cl | ol | - | - | cl | ol | - | cl | - | - | cl |
| 01 08 2134 | 2001 | Ghent (Bel) | Douglas Fluid | - | - | - | - | - | - | - | - | - | - | - |
| 0102 1428 | 2001 | Ghent (Bel) | Sputum | - | ol | - | - | - | - | - | - | - | - | - |
| U93 10957 | 1993 | Ghent (Bel) | Unknown | - | sp | - | - | sp | - | - | - | - | - | - |
| 01 08 4110 | 2001 | Ghent (Bel) | BAL | - | - | - | - | - | - | - | - | - | - | - |
| LUH 7552 | 2001 | Holiday camp (Ned) | Sputum | - | - | - | - | sp | - | - | - | - | ol | - |
| PHLS08959 | 2003 | Liverpool (UK) | Sputum | - | - | - | - | scl | - | - | - | - | - | - |
| PHLS08960 | 2003 | Manchester (UK) | Sputum | - | - | - | ol | scl | - | - | ol | ol | ol | cl |
| PHSL08916 | 2003 | Birmingham (UK) | Sputum | - | - | - | - | - | - | - |  |  | ol |  |

^a^ Abbreviations:

Confluent lysis (cl), opaque lysis (ol), semiconfluent lysis (scl), several plaques (sp), no infection (-).

Hungary (Hun), Australia (Aus); Belgium (Bel), Roumenia (Rom), Germany (Ger), Tunesia (Tun), Turkey (Tur), United Kingdom (UK), The Phillipines (Ph), Bulgaria (Bul), Pakistan (Pak), Congo (CON), Collection Ecole Santé Publique, Anderlecht (CESPA), University Hospital (UH).

^b^ Sputum samples were collected from CF patients.
